# Complete Genome Sequence of a Psychrophilic Bacterium, *Pseudoalteromonas* sp. Strain APM04, Isolated from the Seafloor of the South Mariana Trough, Pacific Ocean

**DOI:** 10.1128/mra.00374-22

**Published:** 2022-07-27

**Authors:** Mahoko Taguchi, Yong Guo, Tomoyasu Nishizawa, Shigeru Chohnan, Yasurou Kurusu

**Affiliations:** a Graduate School of Agriculture, Ibaraki University, Ami, Ibaraki, Japan; b Ibaraki University College of Agriculture, Ami, Ibaraki, Japan; University of Delaware

## Abstract

The complete genome sequence of *Pseudoalteromonas* sp. strain APM04, which is a psychrophilic bacterium that inhabits the seabed of the South Mariana Trough, Pacific Ocean, was determined to characterize the genetic features associated with evolution in extremophilic and oligotrophic deep seawater.

## ANNOUNCEMENT

Psychrophiles found in the deep sea, which remains at 5°C or less throughout the year, are thought to grow very slowly to conserve limited nutritional resources (1–3). Here, we carried out complete genome sequencing of a psychrophilic bacterium strain, APM04, which was predicted to be a member of the genus *Pseudoalteromonas* based on the 16S rRNA sequence; it was obtained from a depth of 2,880 m (4.1 m below the seafloor at the Snail site) on the seafloor of the South Mariana Trough (12°57′N, 143°37′E) during the voyage conducted as a part of the Archaean Park Project (4, 5).

APM04, isolated as a single colony, was grown on 1/2 TZ medium (g/l): Polypepton (Nihon Pharmaceutical), 2.5; Bacto yeast extract (Thermo Fisher Scientific), 0.5; HEPES (Merck KGaA), with Kester’s artificial seawater-1.5% Bacto agar (Merck KGaA) (6), and the APM04 DNA was extracted from multiple colonies grown on the solid plate using a PowerSoil kit (Qiagen). A paired-end library was established using a TruSeq DNA PCR-free kit (Illumina), and a long-read library was prepared using a 1D genomic DNA library kit (Oxford Nanopore Technologies). Then the paired-end library and the long-read library were sequenced on Illumina MiSeq (2 × 150 bp) and GridION instruments, respectively, to generate 2,493,442 reads with a total length of 376,509,732 bases and 424,961 reads with a total length of 3,134,375,494 bases, respectively. Read processing and genome assembly were performed as described previously (7). Briefly, the paired-end reads were processed using Cutadapt v1.11 with the options of –overlap 10, –minimum-length 51, and –quality-cutoff 20 (8), and the Nanopore reads were trimmed using Nanofilt v2.0.0 with the options of –l 1000 and –q 9 (9). The processed paired-end and long reads were assembled and circularized using Unicycler v0.4.6 with default settings (10). The genome sequences were annotated using GeneMarkS-2+ (11), as implemented in the NCBI Prokaryotic Genome Annotation Pipeline (PGAP) v6.1 (12).

The APM04 genome was composed of two chromosomes, i.e., 3,457,294-bp circular chromosome 1 with an average GC content of 39.8%, containing 25 rRNAs (9 of 5S rRNA, 8 of 16S rRNA, and 8 of 23S rRNA) and 102 tRNAs, and 694,804-bp circular chromosome 2 with a GC content of 39.6%, containing 3 rRNAs (1 of rRNA gene operon). Of all protein coding sequences (CDSs) (3,655 CDSs), 3,060 CDSs (83.7%) were found on chromosome 1 and 595 CDSs (16.3%) were present on chromosome 2. The APM04 genome shared average nucleotide identities of 97.90%, 97.77%, 97.74%, 97.50%, and 97.46% with the closest sequenced genomes of *Pseudoalteromonas* sp. strain BSi20480 (GenBank accession number GCF_000241365.1), Pseudoalteromonas marina DSM 17587^T^ (GenBank accession number GCF_000238335.3), Pseudoalteromonas marina 13-15 (GenBank accession number GCF_900141965.1), *Alteromonadales* bacterium TW-7 (GenBank accession number GCF_000169055.1), and Pseudoalteromonas marina ECSMB14103 (GenBank accession number GCF_002407085.1), respectively, which were estimated using JSpeciesWS v3.9.3 (13). The genetic features of the psychrophilic bacterium *Pseudoalteromonas* sp. strain APM04 may help us to infer the evolutionary characteristics in oligotrophic ecosystems and provide genetic information on useful enzymes that function under low-temperature conditions.

### Data availability.

The genome sequences of *Pseudoalteromonas* sp. strain APM04 were deposited in the DDBJ/ENA/GenBank database with the accession numbers CP094441 and CP094442 for the circular chromosomes. The SRA accession numbers are SRR18360445 and SRR18360446 for the MiSeq and Nanopore raw reads, respectively.
